# Effects of Core Size and Surfactant Choice on Fluid Saturation Development in Surfactant/Polymer Corefloods

**DOI:** 10.1021/acs.energyfuels.3c04313

**Published:** 2024-01-26

**Authors:** Andrea Rovelli, James Brodie, Bilal Rashid, Weparn J. Tay, Ronny Pini

**Affiliations:** † Department of Chemical Engineering, Imperial College London, South Kensington, London SW7 2AZ, U.K.; ‡ BP International Ltd, Chertsey Road, Sunbury-on-Thames TW16 7LN, U.K.

## Abstract

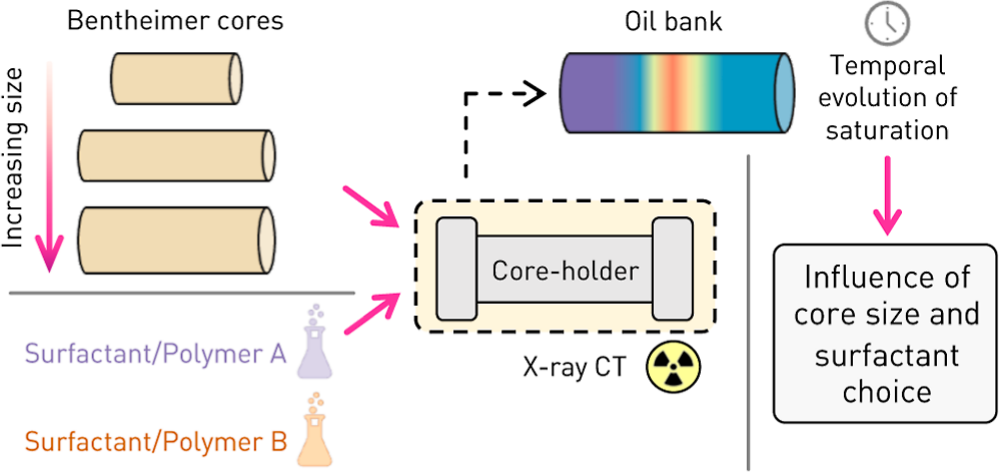

Surfactant/polymer flooding allows for a significant increase in oil recovered at both laboratory and field scales. Limitations in application at the reservoir scale are, however, present and can be associated with both the complexity of the underlying displacement process and the time-intensive nature of the up-scaling workflow. Pivotal to this workflow are corefloods which serve to both validate the extent of oil recovery and extract modeling parameters used in upscaling. To enhance the understanding of the evolution of the saturation distribution within the rock sample, we present the utilization of X-ray computed tomography to image six distinct surfactant/polymer corefloods. In doing so, we visualize the formation and propagation of an oil bank by reconstructing multidimensional saturation maps. We conduct experiments on three distinct core sizes and two different surfactants, an SBDS/isbutanol formulation and an L-145-10s 90 formulation, in order to decouple the effect of these two parameters on the flow behavior observed in situ. We note that the oil production post oil bank breakthrough is primarily influenced by the surfactant choice, with the SDBS/isobutanol formulation displaying longer tailing production of a low oil cut. On the other hand, the core size dominated the extent of self-similarity of the saturation profiles with smaller cores showing less overlap in the self-similarity profiles. Consequently, we highlight the difference in applicability of a fractional flow approach to larger and smaller cores for upscaling parameter extraction and thus provide guidance for corefloods where direct imaging is not available.

## 1 Introduction

Net zero targets have seen widespread commitments^1,2^ and, within proposed energy transition plans, oil and gas are still expected to act as primary energy sources.^3^ So-called primary and secondary oil recovery methods retrieve just 10 and 30% of oil in place, respectively.^4−6^ Enhanced oil recovery (EOR) techniques have always been of commercial and research interest through the decades.^7−9^ Albeit occasionally subject to criticism in regards to its future role in the energy transition^10^—discussion further complicated by nationalization^11^ and politicization^12^—EOR is, importantly, also expected to play a key position in energy security.^13,14^ Among the many available EOR techniques,^15−17^ chemical EOR (cEOR) is often seen as a favorable option from both a recovery and a CO_2_ intensity viewpoint.^18,19^ Within cEOR techniques, surfactant/polymer flooding exhibits excellent recovery potential.^20^ Both microscopic—via the liberation of trapped oil—and macroscopic—via the enhancement in displacement efficiency—recovery factors are greatly improved and recoveries of up to 70% are obtainable.^5^ Despite this theorized efficacy, surfactant/polymer flooding has seen sparse industrial use.^21,22^ A major limiting factor is the incompatibility of the underlying chemicals to harsh reservoir conditions^23^—reservoir conditions that make up 60% of all oil reserves.^24^ Recently, however, through the rapid development of novel surfactants^25^ and polymers,^26^ the technique has proven successful in both harsher conditions^27,28^ and offshore applications.^29^ Dampening the development and implementation of new formulations is the laborious and time-consuming nature of the surfactant/polymer flooding workflow.^30,31^ Pivotal to this process are corefloods which are needed to determine, inter alia, oil recovery, chemical retention and other parameters used in process scale-up.^32^ Interpretation of corefloods is typically solely dependent on effluent analysis;^33^ this, inherently, is a limited viewpoint and can lead to uncertainty about in situ behavior such as emulsification or polymer degradation.^34−36^ To this aim, direct imaging has proven invaluable in developing a greater understanding of both flow and transport within these complex systems.^37^ Microfluidics and micro X-ray computed tomography (CT) have allowed for insights into a range of microscale phenomena such as stability of emulsions^38^ and pore-scale distribution of fluids.^39,40^ However, by nature, these two approaches are restricted in both sample and viewing size^41^ and, as such, are often used as prescreening experiments, or in combination, to corefloods;^42^ promising exception being larger micromodels capturing macroscale behavior successfully.^43^ Medical X-ray CT, on the other hand, allows for imaging of representative core samples and, most commonly, extraction of saturation profiles.^44,45^ Despite this, only rarely have these studies expanded on the direct imaging results, notable cases being the incorporation of internal profiles in evaluating modeling approaches^46^ and the provision of insights into the existence of instabilities in the underlying displacement process.^47^

Surfactant/polymer flooding remains a complex process with many phenomena still currently uncertain and subject to academic and industrial interest—such as flow regimes^48^ and retention.^49^ We contend that multiscale direct imaging approaches^50^ have the greatest potential in aiding the true understanding of the underlying physics of the process; however, each individual imaging approach must be further refined as, currently, insights have been both limited and primarily qualitative. To this aim, within this study, we demonstrate the flexibility and value inherent to medical X-ray CT imaging by investigating the effect of both surfactant choice and core size on the internal dynamics of surfactant/polymer corefloods. The latter of which has, to our knowledge, only been investigated in the scope of oil bank formation.^51^ Through core-flooding experiments, we reconstruct multidimensional representations of the saturation distribution and are able to note the presence, and temporal evolution, of an oil bank. By use of self-similarity profiles, we identify differences in tailing and the applicability of fractional flow theory within the different experiments, factors which we then quantify and attribute an associated dominant cause through the use of an exponential variogram approach. Finally, via the in situ imaging, we highlight important considerations for the interpretation of surfactant/polymer coreflood for the often case where direct imaging is not available.

## 2 Experimental Section

### 2.1 Materials

Surfactant/polymer floods were conducted on cores of three distinct sizes with two different surfactant formulations, totaling six unique experiments. For all experiments, decane (≥95%, Sigma-Aldrich, CAS: 30570) was used as the oleic phase. The aqueous-phase electrolyte concentration was varied utilizing sodium chloride (≥99.5%, Sigma-Aldrich, CAS: 31434-M), which, where a brine solution was used, was equal to the optimal salinity for the particular surfactant formulation. Two formulations of surfactant mixtures were used throughout the experiments: a 3 wt % sodium dodecylbenzenesulfonate (Technical grade, Sigma-Aldrich, CAS: 289957) (SDBS) with 5 wt % isobutanol (99.5%, Sigma-Aldrich, CAS: 294829) as a cosolvent formulation and a 1 wt % L-145-10s 90 (90% active, Sasol) formulation from the ALFOTERRA series. The polymer component of the surfactant/polymer mixture was HPAM (SNF Floerger) with FP3330s being used for the SDBS/isobutanol formulation and FP3530s being used for the L-145-10s 90 formulation, both cases utilizing a polymer concentration of 1500 ppm. The polymers are identical in hydrolysis percentage (25–30%) but differ in molecular weight (8 vs 16 mDa). To displace the surfactant/polymer solutions into the core, light mineral oil (Sigma-Aldrich, CAS: 330779) was used. All experiments used Bentheimer sandstone cores (Kocurek Industries, Inc.) of differing dimensions. The resulting combination of fluid/rock and associated rock properties are given in Table 1.

**Table 1:** Rock Properties and Associated Fluid Combinations Used throughout Experiments 1–6^a^

	rock properties				fluid selection			
exp #	length (cm)	diameter (cm)	ϕ (%)	*K* (D)	surfactant	polymer	NaCl % wt	oil
1	10	3.81	23.9	1.9	A	FP3330s	3.7	decane
2	15	3.81	23.8	2.9	A	FP3330s	3.7	decane
3	15	5.08	23.1	1.3	A	FP3330s	3.7	decane
4	10	3.81	23.6	1.9	B	FP3530s	3.5	decane
5	15	3.81	23.7	2.3	B	FP3530s	3.5	decane
6	15	5.08	22.8	2.0	B	FP3530s	3.5	decane

a Surfactant A refers to the SDBS/isobutanol surfactant formulation, while surfactant B refers to the ALFOTERRA surfactant solution. Porosity was calculated from the combination of X-ray CT images, and permeability was measured experimentally.

### 2.2 Experimental Apparatus

**Figure 1:**
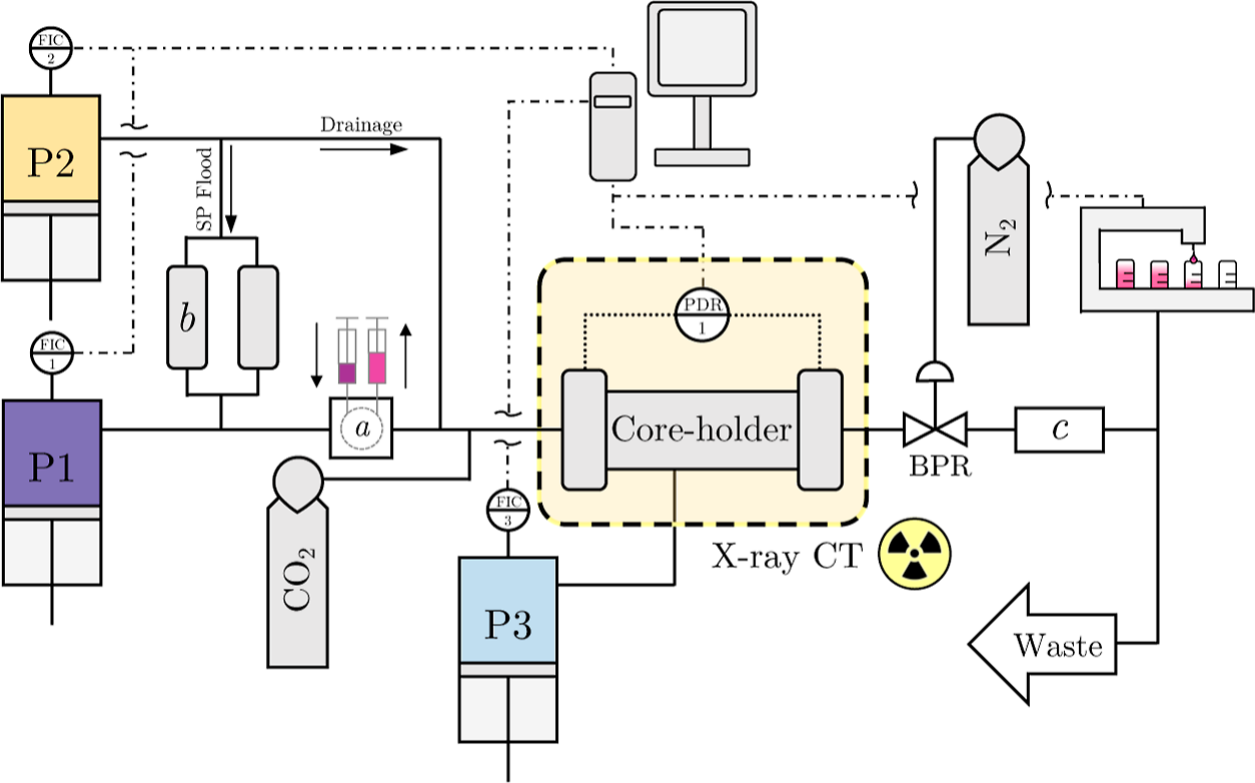
Simplified representation of core-flooding setup used for all experiments. Additional to the labeled highlighted components are the injection system (a), samples vessels, (b) and conductivity meter (c). A detailed P&ID for this setup is presented in the Supporting Information.

The experimental system for all core-flooding experiments is shown in Figure 1 and is an extension of that of Kurotori et al.^52^ to allow for multiphase experiments. The setup can be subdivided into two main sections: injection and downstream, based on their relative position to the core holder. The core holder itself is custom built with an aluminum barrel—to minimize beam hardening artifacts^53^—and titanium end-caps, between which, core samples of either 3.81 or 5.08 cm diameter and either 10 or 15 cm length are housed for the experiments. The end-caps also feature a grooved spiderweb design to aid fluid distribution on the core face upon injection. To prevent bypass, samples are wrapped in two layers of heat shrink tubing with a confining pressure set via a piston syringe pump (P3, 500D Teledyne ISCO). The core holder is housed horizontally in a Toshiba Aquilion 64 TSX-101A X-ray CT scanner, and both differential and absolute pressures are measured with a differential pressure transducer (PDR-1, 3 bar PRD-33X Keller UK); this, along with all pump flow rates and pressure, is continuously logged via a computer. On the injection side, two pumps, an aqueous-phase pump (P1, 1000D Teledyne ISCO) and an oleic-phase pump (P2, 1000D Teledyne ISCO), allow for uninterrupted flow for the duration of the experiments. Two items, a dual-position six-port valve (a, Cheminert six-port injection valve, Vici) and sample vessels (b, 304L SS Sample Cylinder, Swagelok) allow for tracer solutions and surfactant/polymer slugs to be injected, respectively. Downstream, a backpressure regulator (BPR, ZF series Equilibar) and nitrogen cylinder are used to hold the pore pressure of the core at a chosen set-point. Last, a conductivity cell and associated meter (c, model 8032, Amber Science) in addition to an in-house-built fractional collector^54^ allow for outlet collection and measurements.

### 2.3 Experimental Methodology

#### 2.3.1 Solution Preparation and Characterization

Both brine and surfactant/polymer solutions were prepared using 18 MΩ deionized water and, for the surfactant/polymer solutions, were stirred for at least 48 h. Both solutions were filtered, the former utilizing 0.45 μm pore size filters and the latter utilizing 1.20 μm pore size filters (MF-Millipore membrane filters, Sigma-Aldrich, CAS: HAWP04700 & RAWP04700). For the surfactant/polymer solutions, a filtration ratio was also calculated in order to ensure a value of approximately 1. Viscosity measurements were performed at 25 °C in a rotational rheometer (Haake MARS rotational rheometer, Thermo Fisher) equipped with a double-gap cylinder geometry with shear rates ranging from 1 to 1000 s^–1^.

#### 2.3.2 Phase Tests and Salinity Screening

To determine both the optimal surfactant combination and the associated optimal salinity, a series of phase tests were performed. Phase tests consisted of the preparation of a surfactant solution at specific surfactant concentration and, if needed, a chosen cosolvent concentration. Using a pipet (±0.03 mL), 5 mL of this solution was then introduced into a test tube where a varying amount of NaCl was added as to sweep a range of salinities. The oleic phase was then similarly introduced, and the test tubes were mixed and allowed to equilibrate for at least 3 d. Once equilibrated, the size of a middle microemulsion phase, if present, was measured to then gauge the efficacy of the surfactant/polymer mixture in lowering the interfacial tension.

Salinity screening tests were also performed to ensure the stability of the surfactant solutions at the chosen optimal salinities. These were performed identically to the phase tests, with the only difference being the exclusion of the oleic phase, and were judged by ensuring that the surfactant mixture did not precipitate and was clear.

#### 2.3.3 Coreflooding

Experiments were performed at room temperature (approximately 22 °C), and all cores were dried for 72 h at 65 °C prior to being mounted in the core holder. Throughout the experiment, an overburden pressure of 30 bar was held with water using the confining pressure pump to prevent bypass. The core was first flushed with CO_2_ (≥99%, BOC), subsequently, brine was injected for an excess of 10 PV to ensure saturation. The pore volume pressure was then raised to 8 bar via the back-pressure regulator, and permeability measurements were conducted by varying the inlet flow rate and measuring the corresponding pressure drop on the core. Drainage was then performed via the continuous injection of decane for at least 10 PV. Once connate water saturation was achieved, waterflooding was commenced with the injection of brine, completion of which was determined by ensuring that the pressure drop across the core was steady. Subsequently, the surfactant/polymer mixture was injected continuously for ≥1.5 PV—via a gravity favorable displacement where mineral oil displaces the surfactant/polymer solution housed in the sample vessels, followed by another waterflood until termination of the experiment.

From the first waterflood to the termination of the experiment, the injection flow rate was constant and dependent on the core diameter to achieve a consistent frontal advance rate between all experiments. The frontal advance rate chosen was 7 ft/d and is a trade-off between field scale—typically ranging between 1 and 28.5 ft/d for oil recovery and near well injection respectively^55^—and practical, time-wise, for the experiment, flow rates. The flow rate was also selected as to ensure that the critical capillary number was surpassed solely due to the reduction in interfacial tension from the presence of the surfactant and not due to the increase in viscosity from the introduction of the polymer.^56^ Thus, the resulting flow rates were 0.39 and 0.70 mL min^–1^ for the 3.81 and 5.08 cm diameter cores, respectively.

### 2.4 Image Processing and Analysis

The X-ray CT scanner, Toshiba Aquilion 64 TSX-101A, was operated with a radiation energy level of 120 kV and a tube current of 200 mA; the resulting field of view was (512 × 512) voxels with sizes of (0.122 × 0.122) mm^2^ in the transverse directions and 1 mm in the longitudinal direction relative to the core alignment. Scans were taken approximately every 0.07 PV—5 or 7 min depending on the core size—and the scanning time itself was between 7 and 10 s. Given the low flow rate and the fast scanning time, the internal displacement process is assumed static during a scan.

The raw images were subsequently processed utilizing an in-house MATLAB workflow in order to extract both porosity and saturations. This process involves using the linear combination of scans.^57^ Voxel porosity, ϕ, is calculated via the combination of a dry core scan and a water-saturated core scan, as follows
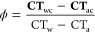
1Here, **CT**_wc_ is the CT number vector for all voxels in a water-saturated core, **CT**_ac_ is the CT number vector for all voxels in an air-saturated core, CT_w_ is the CT number for water in a core holder, and CT_a_ is the CT number for air in a core holder. Voxel oil saturations, ***S***_o_, on the other hand, are calculated as follows^58^
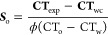
2Here, **CT**_exp_ is the CT number vector for all voxels in an experimental scan and CT_o_ is the CT number for the oil in a core holder. For all experiments, pure component CT numbers were measured by scanning sample tubes held within the core holder, and mean values and associated confidence intervals are reported in the Supporting Information.

Through the image processing, the voxels were coarsened to achieve a reasonable error in the associated properties. By applying a standard error propagation technique to eqs 1 and 2, uncertainties for both porosity and saturations under different coarsening schemes can be calculated.^59,60^ Coarsening is also necessary to ensure that the voxel errors are not autocorrelated,^61^ and both the resulting lag plots and derived equations for error propagation are given in the Supporting Information. Following this approach, three-dimensional representations were reconstructed utilizing (2.93 × 2.93 × 3) mm^3^ voxels, while two-dimensional representations were reconstructed using (1.95 × 1.95 × 2) mm^3^ voxels. This yields relative errors of 0.6 and 0.2% in the porosity and 8.8 and 3.5% in the saturation, respectively. One-dimensional representations due to sampling over whole image slices were found to have negligible errors. Last, two- and three-dimensional visualizations presented within this work were generated using a framework built upon the MATLAB Reservoir Simulation Toolbox.^62^

## 3 Results

### 3.1 Solution Characterization

**Figure 2:**
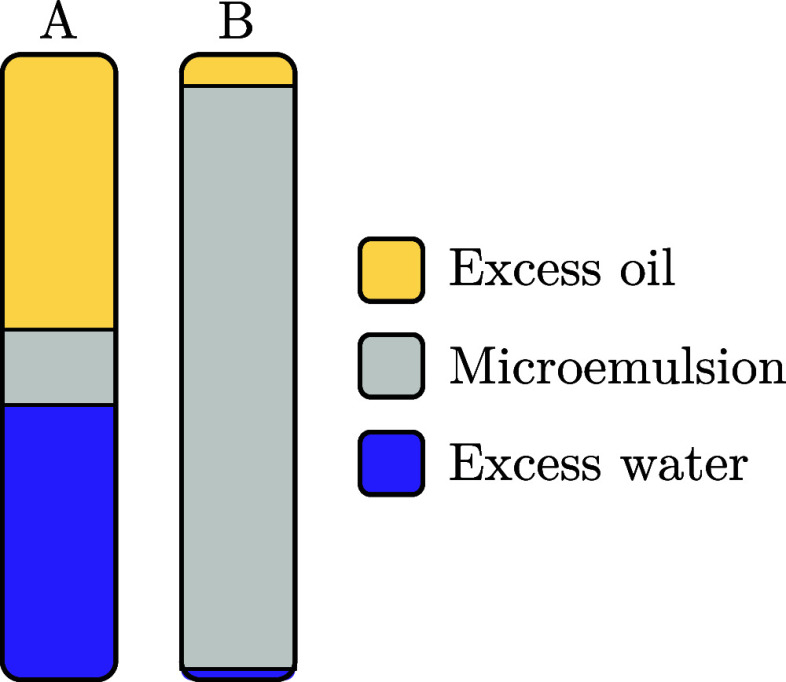
Simplified illustration of phase test results for both surfactant solutions at optimal salinity—shown to scale. Surfactant A refers to the SDBS/isobutanol solution, while surfactant B refers to the L-145-10s 90 solution. The corresponding optimal salinities were 3.7 and 3.5% wt NaCl. The associated photographic version of results is available in the Supporting Information.

Pivotal to the overall performance of a surfactant/polymer flood is the surfactant’s ability to significantly reduce the interfacial tension to ultralow values. This can be inferred via phase tests and through the presence and size of a microemulsion. Figure 2 illustrates how, with both surfactant formulations, a middle microemulsion phase is formed.

Evident from Figure 2 is a significant difference in the sizes of the microemulsions. Applying Huh’s equations^63^ leads to approximate IFT values of 1.5 × 10^–2^ and ≤1 × 10^–3^ mN m^–1^ for surfactant formulations A (SDBS/isobutanol) and B (L-145-10s 90), respectively—results aligned with the available literature.^64,65^ Despite the large relative difference in predicted IFT values, both are sufficient to overcome the critical capillary number and thus liberate previously trapped oil. Notable for successive result interpretation is that formulation B is a comparatively better surfactant formulation to A. Both surfactant formulations were also found to be stable at their, respective, optimal salinity—opaqueness, not cloudiness, present with the addition of the polymer. Last, viscosities were tuned by varying the polymer concentration in order to target 25 mPa s (additional details are provided in the Supporting Information).

### 3.2 Coreflooding

The surfactant/polymer corefloods were operated as tertiary recovery methods; as such, the initial water saturation differed between the six experiments. These, along with the PV injected of surfactant/polymer solutions, are given in Table 2.

**Table 2:** Initial Water Saturations, *S*_w_^*i*^, and Surfactant/Polymer PV Injected for the Six Experiments Considered^a^

exp #	surfactant	*S*_w_^*i*^	PV injected
1	A	0.64	2.1
2	A	0.63	2.0
3	A	0.59	1.6
4	B	0.63	2.1
5	B	0.61	2.1
6	B	0.62	1.7

a Surfactant A refers to the SDBS/isobutanol surfactant mixture, while surfactant B refers to the ALFOTERRA surfactant solution.

**Figure 3:**
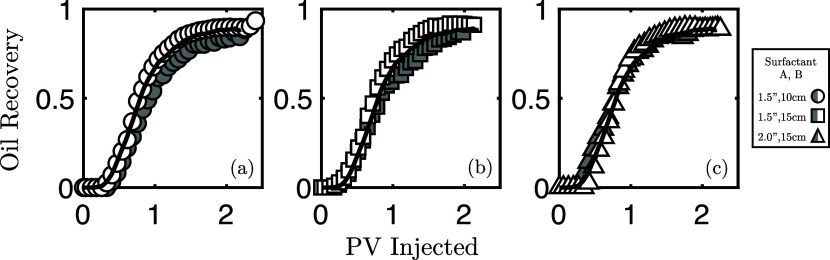
Oil recovery profiles for the six experiments presented with mean profiles shown as a solid line. Marker symbols and subplots (a–c) refer to different core sizes, while marker color refers to the different surfactant formulations. Oil recovery computed from saturations extracted from the X-ray CT images.

Minor variations are seen in the initial conditions of the experiments and are a result of the multistep, and multiday, nature of the preceding core-flooding experimental steps, as outlined in Section 2.3.3. The pore volumes of surfactant/polymer injected also differ with regards to the two larger cores (experiments 3 and 6); this, however, has an insignificant effect on the performance of the corefloods as the minimum slug size is considerably surpassed in all experiments, evident from examining the oil recovery profiles, Figure 3, where plateauing occurs at ≈1.5 PV.

Figure 3 also presents a first perspective on the differences between the experiments considered. Despite the approximately identical ultimate recovery, it is clear from the temporal profiles that dissimilarities are present, and both surfactant formulation and core size influence the recovery process—observation that can be further investigated via the use of in situ imaging.

#### 3.2.1 X-ray Imaging

**Figure 4:**
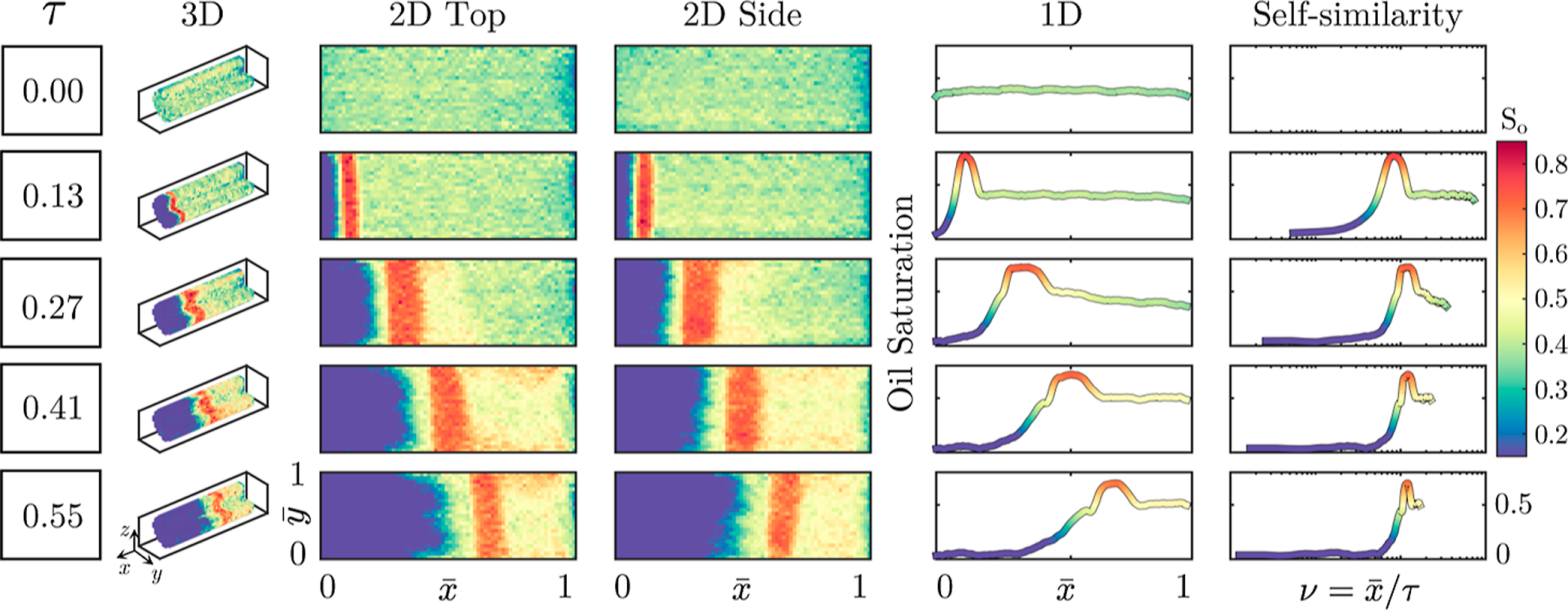
Exemplary visualizations of surfactant/polymer flood in a 5.08/15 cm core imaged via X-ray CT (experiment 6). Rows refer to different dimensionless times, columns to different possible representations, and color scheme to oil saturation. For all representation, flow is from the left side of the visualization to the right. Two-dimensional representations are split in “top” and “side” corresponding to compressing the three-dimensional core from the top and side to generate two-dimensional images, respectively.

Figure 4 illustrates exemplary reconstructions obtainable via X-ray CT imaging for experiment 6. Shown are multidimensional representations of the surfactant/polymer flood at differing experimental time steps, denoted by the pore volume injected, τ = (*Q*·*t*)/PV_core_, where *Q* is the volumetric injection flow rate.

**Figure 5:**
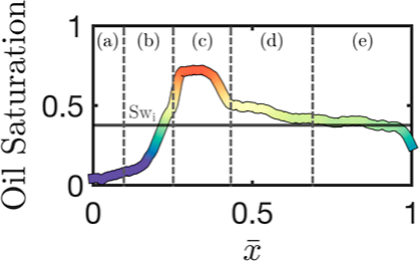
Exemplary internal saturation profile of a surfactant/polymer flood in a 5.08/15 cm core computed via X-ray CT (experiment 6). Highlighted are five different regions (a–e): (a) final saturation, (b) oil bank trailing edge, (c) oil bank, (d) fast moving oil, and (e) residual saturation.

From the multidimensional representations in Figure 4, we note the formation and propagation of a region of high oil saturation, an oil bank. This is a characteristic feature of surfactant/polymer floods and is indicative of a successful flooding process as it can be associated with the ability of the surfactant to lower the interfacial tension sufficiently such that trapped oil is liberated.^51^ Examining the internal profiles, we also note a homogeneous initial saturation profile at an intermediate saturation value due to the operation of the surfactant/polymer flood as a tertiary recovery method, as a result of the inherent homogeneous nature of Bentheimer. These different regions, and others of interest, can be illustrated, as shown in Figure 5; where a typical one-dimensional internal saturation profile subdivided into different areas is shown.

**Figure 6:**
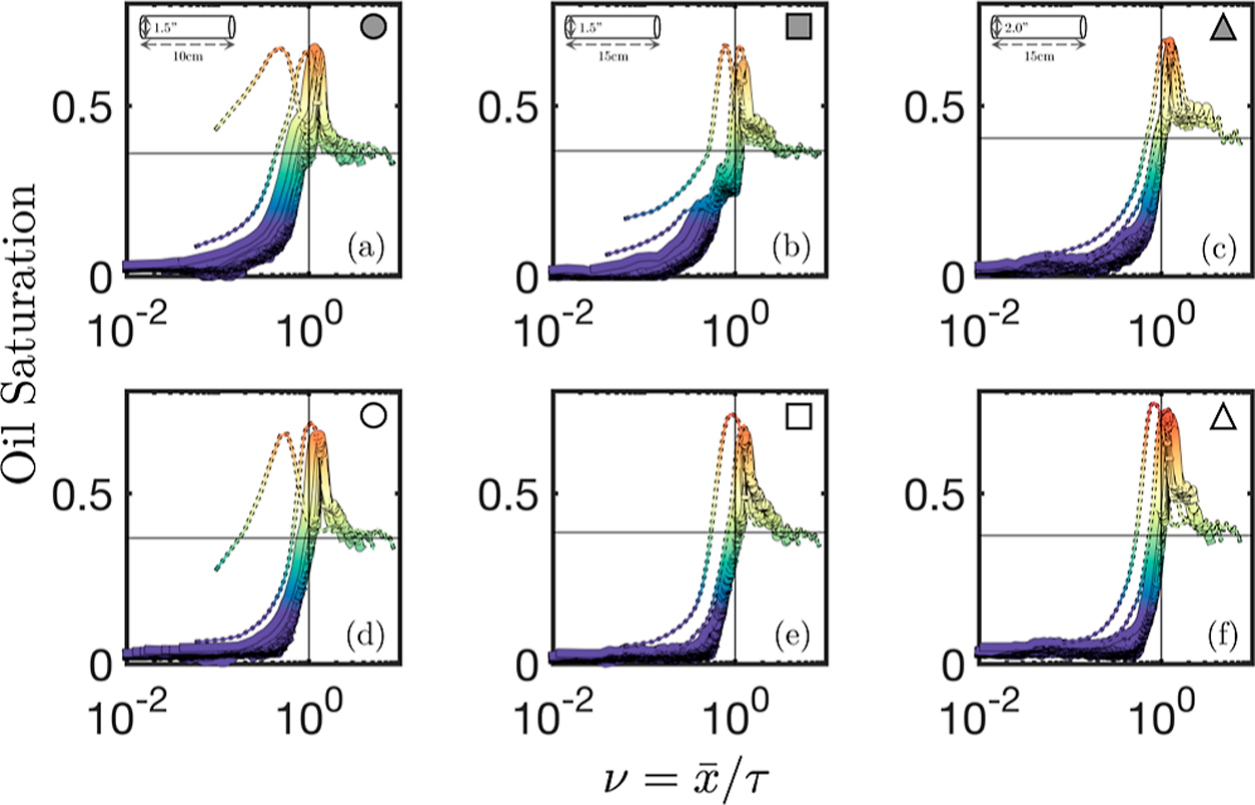
Self-similarity profiles for all six experiments being considered. Row 1 refers to experiments conducted with SDBS as a surfactant and row 2 refers to those performed with the ALFOTERRA surfactant mixture. Each column refers to experiments performed in identical core sizes [D–L]: [3.81–10 cm], [3.81–15 cm], and [5.08–15 cm] in columns 1, 2, and 3, respectively.

From Figure 4, of particular interest is the oil bank appearing to move equidistantly between frames, and given that the frames shown are also equidistant in time elapsed, it can be inferred that the oil bank is moving linearly at a constant velocity. To further investigate this, also shown are self-similarity profiles for the different time steps.^66^ By self-similarity, we refer here to a prominent feature of Riemann problems, whose solution stretches in space and time but does not change shape. Riemann problems are defined by piecewise constant initial data and have formed the basis of the analysis of both waterflooding and polymer flooding.^5,67^ Here, internal profiles are recast in terms of a dimensionless velocity, ν = *x*/τ, allowing one to compare characteristic velocities of different regions of the saturation profiles. From the self-similarity profiles, we can thus note the aforementioned: the high oil region of the internal profile, oil bank, takes on a constant ν value between sequential frames—a constant velocity. Similar behavior can be seen for all regions (regions described in Figure 5) of the internal profiles; this implies not only linear displacement velocities but also, given the equal differences in constant velocities, linear growth of the regions within the internal profiles. Self-similarity profiles thus allow one to capture a significant amount of information for surfactant/polymer floods and are a convenient avenue to compare multiple experiments; as such, Figure 6 presents the self-similarity profiles for experiments 1–6 (a–f) for all experimental time frames considered.

From Figure 6, we note differences between the self-similarity profiles of the six experiments considered; specifically, major distinctions can be seen in the degree of profile overlap, the presence of profile tailing, and in the oil bank characteristics.

#### 3.2.2 Linear Propagation of Displacement Process

For a Buckley–Leverett-type displacement, the self-similarity profiles can be shown to collapse onto a singular, characteristic, curve. This is a direct result of the inherent linear displacement velocities underlying a fractional flow solution; as such, quantifying the degree of misalignment in the self-similarity profiles is an analogue for the deviation of the observed displacement profile from this idealized scenario. The degree of overlap can be quantified by considering the temporal cumulative sum of the area between consecutive self-similarity profiles
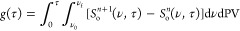
3Here, the integration limits ν_0_ and ν_f_ are computed as the extremes of {ν^*n*^ ∩ ν^*n*+1^} to ensure a bounded area. For a perfect overlap, *g*(τ) = 0, on the other hand, *g*(τ) > 0 implies a deviation from a linear growth and linear displacement process. To best compare the degree of deviation and associated time scale of deviation growth, we utilize an exponential variogram defined as follows
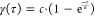
4

Both the sill, *c*, and the effective range, λ = 3*a*, are extracted via the fitting of γ(τ) to the experimentally calculated *g*(τ) using an adapted version of variogramfit^68^ allowing for both optimal parameter extraction but also associated 95% confidence intervals.

**Figure 7:**
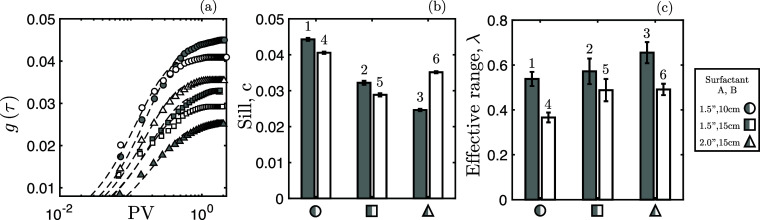
(a) Evaluation of eq 3 for the six experiments considered (markers) and associated fit for eq 4 (dashed lines). Resulting sill value (b) and effective range (c) for variogram fit—eq 4—of experiments considered (labeled 1–6 as per Table 1). For both extracted parameters, 95% confidence intervals are also shown.

Figure 7 presents the experimentally computed *g*(τ) values (a) and the associated fitted sill (b) and effective range values (c) upon application of eq 4. From the sill, we note a clear influence of the core size for both surfactants. Smaller cores display a comparatively larger sill value—ultimately, translating into a distancing from linear growth, and propagation, of different regions within the internal saturation profiles. Notable exception to this trend is experiment 6 where the sill value is considerably larger than the corresponding experiment with surfactant formulation A and both experiments with the intermediate core size (2 and 5). This inconsistency can be best understood by examining Figure 4, where, both in the two- and one-dimensional representations, a fast moving region of mobilized oil can be seen moving ahead of the formed oil bank - region (d) in Figure 5. This region quickly transverses the core, but its presence is significant enough such as to lead to a large increase in the value of *g*(τ) and is not seen as prominently in all other experiments.

On the other hand, from Figure 7c, within a 95% confidence interval, the effective range appears weakly sensitive to the core size—implying that the PV time taken to reach linear growth is approximately the same in all core sizes. The surfactant remains a contributing factor, and overall this implies that the overcoming of any inlet effects before an oil bank is formed is insensitive to the core size but affected by the surfactant choice, with the better surfactant accelerating the process.

#### 3.2.3 Delayed Production via Tailing at Rear of Oil Bank

From Figure 6, differences in the rear of the self-similarity profiles were highlighted. This rear portion of the profiles corresponds to the internal saturation post oil bank breakthrough, and its behavior is directly related to the change in saturation as the profile approaches the final chemical residual oil saturation. The tailing of the saturation profiles is thus associated with an oil recovery period post oil bank breakthrough, a period characterized by a lower oil cut and intermixing with the chemical agents, undesirable due to the need for additional separation. As such, minimizing both the absolute extent and the time frame of the tailing is often most attractive. To capture the transient process in the saturation profiles, the area between each saturation profile post oil bank breakthrough and the final saturation profile of the completed experiment (post chase-waterflood), profiles at chemical residual saturations, is calculated and the resulting deviation is collectively summed, mathematically
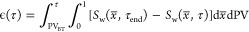
5

**Figure 8:**
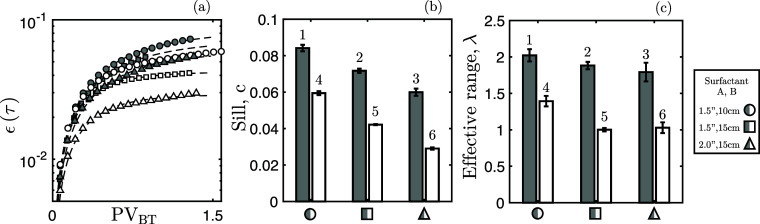
(a) Evaluation of eq 5 for the six experiments considered (markers) and associated fit for eq 4 (dashed lines). Resulting sill value (b) and effective range (c) for variogram fit—eq 4—of experiments considered (labeled 1–6 as per Table 1). For both extracted parameters, 95% confidence intervals are also shown.

Figure 8a presents both the evaluation of eq 5 to the experimental data set and the corresponding fit of eq 4; the associated optimal parameters for both the sill and effective range are then given in Figure 8b,c, respectively.

The sill captures the absolute extent of tailing, and as the recovery between experiments is comparable (see Figure 3), an indication of the amount of oil produced under unfavorable conditions can be garnered. Examining Figure 8b, we note that the extent of tailing is influenced by both the surfactant choice and the core size, with smaller cores exhibiting greater tailing. Combined with the effective range, Figure 8c, we note that both characteristics of tailing are more strongly influenced by the surfactant choice. This can be accounted for by the formulations’ differences in ability to reduce interfacial tension and, subsequently, ability to decrease capillarity effects, notably capillary end-effects.

#### 3.2.4 Oil Bank Characteristics

**Figure 9:**
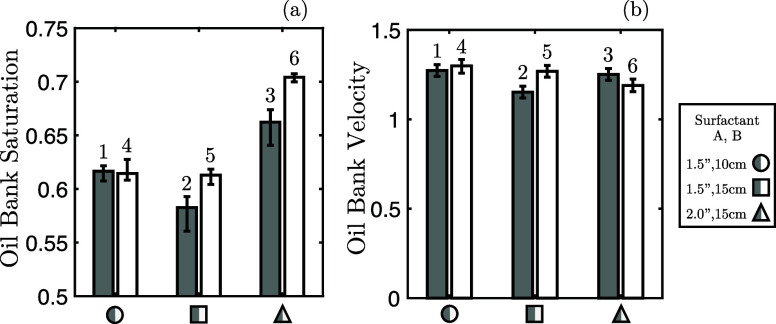
(a) Oil saturation of oil bank for six experiments considered. (b) Characteristic velocity of oil bank for six experiments considered (labeled 1–6 as per Table 1). Associated standard deviation of both parameters are also shown.

The profiles in Figure 6 all exhibited an oil “peak”; this region of the profiles corresponds to the oil bank formed from the surfactant/polymer injection. Given its importance as a desirable attribute, presented in Figure 9a,b are the oil bank’s mean saturation and oil bank’s characteristic velocity, respectively, computed by considering points in the saturation profiles with saturation values in the top 4%.

Examining Figure 9a, we note that the surfactant choice, and thus its efficacy, is the driving mechanism in influencing the oil saturation within the oil bank, with higher oil saturations and thus oil cuts, seen in the experiments performed with the higher-performance surfactant. As mentioned earlier, given similar recoveries between experiments, this result is interconnected with that from Figure 8b as, with less oil produced in the bank, more oil is produced via tailing.

On the other hand, the characteristic velocity of the oil bank, Figure 9b, appears invariant to both the surfactant choice and the core size. As such, the velocity of the oil bank must be a property dominated by either the core properties (porosity or permeability) or the fluid properties (relative permeabilities or capillary pressure). However, given the use of both identical fluids and identical core types in the two sets of differing surfactant experiments, the relative permeability curves and capillary pressure curve are expected to also be an identical intraset. When considered with the differing saturations within the oil banks, and, subsequently, different fluid properties within the oil bank, the core type and its properties are the most probable determinant factor in the velocity of the oil bank—similar observation to that of van Batenburg et al.^66^

## 4 Discussion

### 4.1 Applicability of a Fractional Flow Approach

Despite their simplicity, fractional flow solutions to the two-phase immiscible displacement process are commonly used to quickly interpret experimental results and, via fitting, extract fluid and rock parameters. Extended to chemical flooding, the fractional flow approach utilizes two fractional flow curves—an oil/water curve and a “conservative tracer” curve^69^ defined as
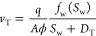
6Here, *v*_T_ is the frontal velocity of the tracer, *q* is the flow rate, ϕ is the porosity, *A* is the cross-sectional area of the rock, *f*_w_ is the fractional flow curve (function of water saturation, *S*_w_), and *D*_T_ is the adsorption constant for the tracer. Inherent to this approach are a significant number of assumptions (outlined in the Supporting Information), and despite this, when applied to surfactant or polymer-flooding experiments, credible results can often be obtained. For more complex scenarios, however, as in surfactant/polymer flooding, the standard fractional flow approach might prove insufficient at capturing the observed behavior. To account for this, Matsuura et al.^46^ proposed an extended fractional flow approach which—for their case to assign different adsorption constants—utilizes three fractional flow curves: a surfactant, a polymer, and an oil/water curve; the two former being described by eq 6.

**Figure 10:**
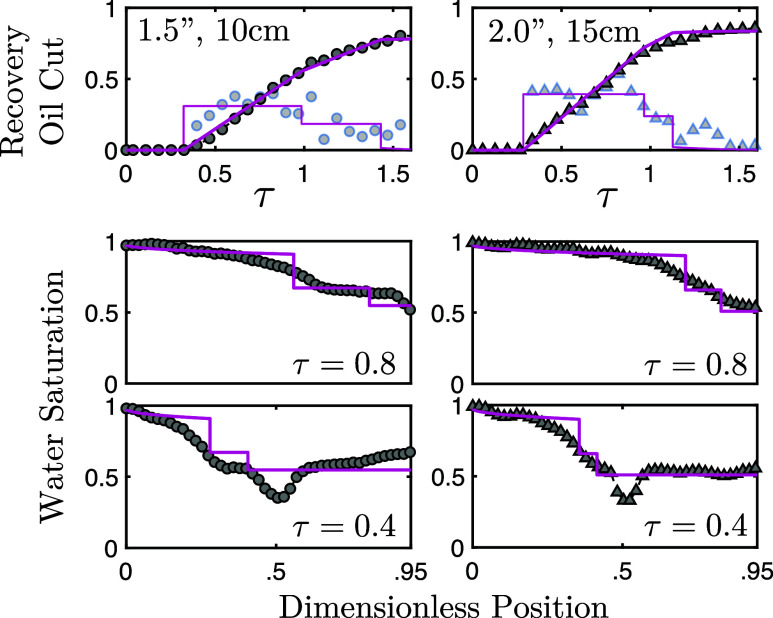
Illustrative difference between fractional flow technique applied to outlet matching (top) in a small core (left) and a large core (right). Late stage (middle) and early stage (bottom) internal water saturation profiles for both experimental, markers, and fractional flow approach, solid line, are also shown.

As mentioned in Section 3.2.2, the degree of overlap of the self-similarity profiles is an important factor when considering the applicability of the fraction flow approach to interpret outlet results and, following, infer internal flow dynamics. To best illustrate this, Figure 10 presents the application of the extended fractional flow approach to experiments 1 and 3—experiments which displayed the highest and lowest degree of self-similarity profile overlap, respectively.

**Figure 11:**
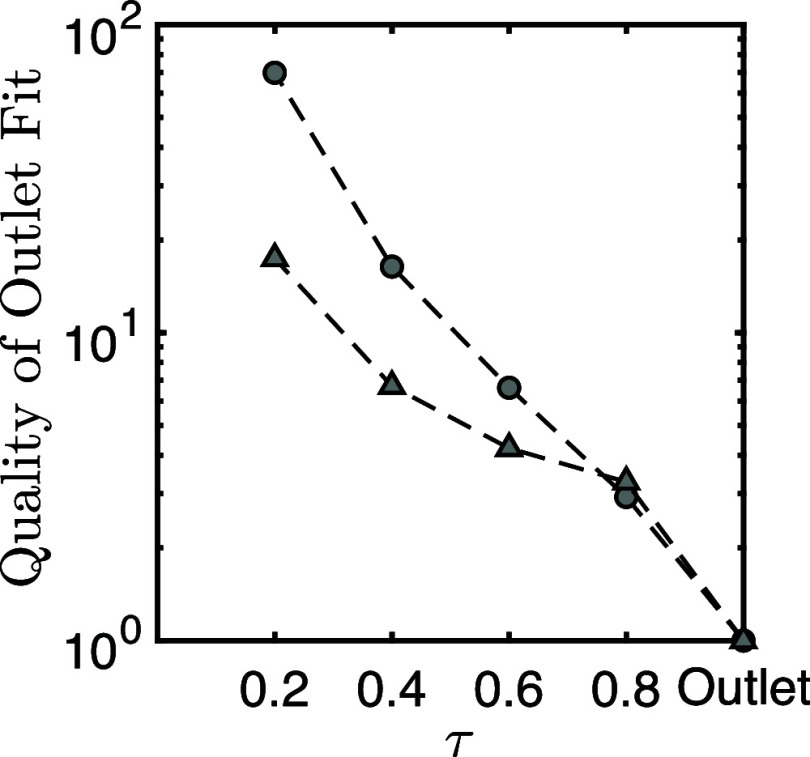
Numerical representation of comparison in quality of fractional flow fits from Figure 10. Quantification of “quality” is normalized such that the outlet fit is equal to 1 and internal profiles are thus multipliers. Absolute values of fits are comparable at 1.4 × 10^–3^ and 2.0 × 10^–3^ for the smaller and larger core, respectively—calculated with eq 7.

In Figure 10 (top), the extended fractional flow was applied to match the outlet oil cut—experimentally determined via sampling—and the associated oil recovery, in order to extract rock and fluid parameters for the experiments (additional details are provided in the Supporting Information). These parameters and the fractional flow model were then used to calculate the associated saturation profiles at two different time points: τ = 0.8 (middle) and τ = 0.4 (bottom). This was done for both experiments: experiment 1 (left) and experiment 3 (right). As expected, when differences in the aforementioned deviation from linear propagation between experiments and the inherent constant velocity solution for fronts within a fractional flow approach are considered, examining the temporal evolution of the fitted model solution shows clear differences between the two experiments. Despite both outlet fits appearing qualitatively representative, as we progress further, time-wise, from the outlet—late stage profile to early stage profile -- the quality of the information captured by the fractional flow model decreases. We also note that, in the case of higher sill value, lower self-similarity profile overlap, the fit degrades comparatively more. To further capture this difference, we can quantify the quality of the fit (QF) at the different time stages via the following equation
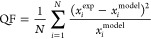
7where *x*_*i*_ are the observations, oil cut/recovery or saturation profiles, experimentally determined (exp) or calculated via the fractional flow approach (model). Computing eq 7 for a range of time steps, and normalizing by the outlet quality of fit, a comparison of the applicability of the fractional flow theory can be made, Figure 11.

Figure 11 presents a normalized visualization of the change in QF as we progress from outlet to late- to early-stage profiles; where the QF reported for each time step are multipliers of the QF at the outlet, meaning a higher value implies a degradation of the fit. Once again, the difference between the two experiments considered is significant (note the logarithmic *y* scale) and, as was previously reported, the degree of overlap within the self-similarity profiles is thus a strong determinant on the applicability of the fractional flow approach. To last appreciate this, one can consider that, in a case where the self-similarity profiles perfectly collapse, the quality of the fit would be invariant at any time stage considered.

Given its ease of use, fractional flow approaches are commonly used to interpret outlet data and extract fluid parameters; fluid parameters which can then be used directly for larger-scale simulations or as initial values for optimization problems utilizing more rigorous flow models. We show that, despite the outlet match appearing successful in both experiments considered, care should be taken in this approach as, although an oil bank might have formed experimentally, self-similarity of the internal profiles is not ensured—a problem which is emphasized in smaller cores but is also surfactant choice-influenced; see Section 3.2.2. As such, where direct imaging is not available to extract internal saturation profiles and verify the degree of overlap in self-similarity profiles, the use of larger cores is always recommended when the extraction of fluid parameters is key. This is in spite of the associated downsides of larger pore volumes, and thus fluids needed, and longer experiments, assuming a constant frontal advance rate.

### 4.2 Versatility of X-ray CT Imaging

The results in Section 3.2.1 focused on displaying the variety of results that can be extracted via X-ray CT imaging. Not highlighted was the versatility and utility these insights can bring. For scenarios with more complexities—presence of heterogeneity^70^–or harsher conditions—requiring a salinity gradient^71^—maximizing the amount of information garnered during a coreflood is pivotal as their effects can often be contradictory. Decoupling the source of the observed behavior is, however, necessary in order to correctly model and, subsequently, scale the experimental result. Viewing the internal dynamics of the displacement process can thus help elucidate features that might be ambiguous by solely analyzing the outlet samples.

**Figure 12:**
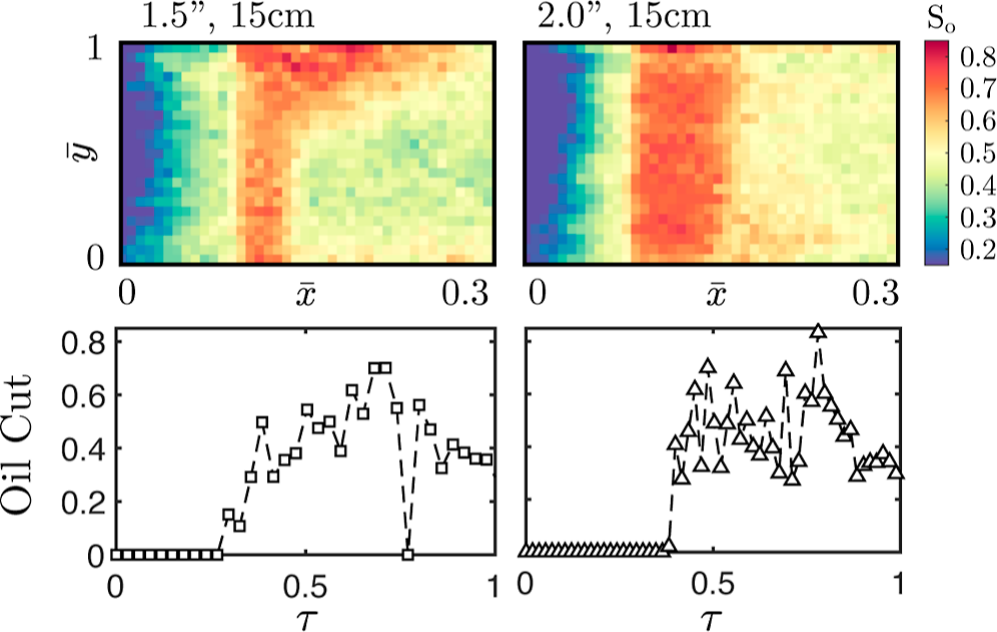
Side view of two-dimensional saturation maps and associated oil cut sampled at the core outlet for experiments 5 (left) and 6 (right). Notable is the difference in extent of gravity effects and associated effect on the oil cuts measured.

Figure 12 illustrates this concept by comparing the in situ profiles for experiments 5 and 6 and the corresponding oil cuts measured at the outlet. Evident from the internal saturation maps is the difference in the extent of gravity effects between the experiments. This effect then directly influences the shape of the oil cut profiles measured at the outlet. Experiment 6 (right) displays a fast and sharp rise in the oil cut, in contrast with experiment 5 (left) which shows a slow and gradual rise. This result is intuitive as, with gravity effects influencing the shape of the oil bank, a comparatively lower saturation is expected with the breakthrough of the leading edge of the deformed bank. Despite the intuitive nature, without the internal saturation maps, determination of the cause for the resulting shape of the oil cut profiles would be uncertain. In the case presented here, modeling experiment 5 would require at least a two-dimensional model to capture buoyancy differences; however, had the cause for the oil cut profile shape been different, the additional dimension in the model would be both an unnecessarily added computational cost and an added complication.

It is clear that direct imaging can thus not only probe fundamentals of the flow dynamics, as was done in this work, but also help guide the iterative, and time-consuming, nature of the surfactant/polymer workflow by both identifying issues with performed corefloods, allowing rapid rectification and tuning, and reducing the uncertainty in fluid parameters used for up-scaling.

## 5 Conclusions

In this work, we successfully applied X-ray CT imaging to visualize the flow dynamics within six surfactant/polymer corefloods. Bentheimer cores of three different sizes and two differing surfactant formulations were tested to investigate their corresponding effects on the surfactant/polymer flooding process. The surfactant/polymer floods were operated as tertiary recovery methods, and in all experiments performed, oil recoveries in excess of 90% were observed. Through the use of direct imaging and the reconstruction of the internal saturation profiles, the formation of an oil bank was also noted for all cases considered, indication of a successful surfactant/polymer system. Examining the experimentally derived self-similarity profiles, we highlighted the following.

- Extent to which the displacement process scales linearly is strongly dominated by the core size.
- Time taken for profiles to reach self-similarity is relatively unaffected by the core size but affected by the surfactant choice.
- Oil production post oil bank breakthrough is primarily influenced by surfactant choice; effect of the core size is still notable.
- Oil bank velocity is invariant to the core size, surfactant choice and, notably, oil saturation.

Last, we emphasized the additional advantages that the in situ imaging provides in both helping guide modeling approaches and offering a viable avenue for experimental diagnosis. These two factors can significantly accelerate the iterative nature of the overall process up-scaling workflow, ultimately aiding in the deployment of cEOR techniques in more unconventional and harsher reservoirs. We suggest that future studies further demonstrate the advantages of direct imaging by investigating the application of the explored methodology to cores with structured heterogeneities, such as layers or fractures, as this could prove pivotal in aiding the development of the fundamental knowledge needed to more accurately model the surfactant/polymer flooding process at a field scale.

## Supporting Information Available

Detailed experimental setup P&ID; pure component CT numbers; derivation of X-ray CT error and associated application to 3D and 2D representations including error correlation; solution characterization; fractional flow model assumptions; and fractional flow application (PDF)
