# Supplementary material for: Effects of Core Size and Surfactant Choice on Fluid Saturation Development in Surfactant/Polymer Corefloods
Source: Energy Fuels. 2024 Jan 26;38(4):2844–54. doi: 10.1021/acs.energyfuels.3c04313 (PMC10875641; doi:10.1021/acs.energyfuels.3c04313)
Supplement: Supplementary file 1 — ef3c04313_si_001.pdf [file ef3c04313_si_001.pdf]

Supporting Information for 'Effects of Core Size and Surfactant Choice on Fluid Saturation Development in Surfactant/Polymer Corefloods'

Andrea Rovelli,[†] James Brodie,[‡] Bilal Rashid,[‡] Weparn J. Tay,[‡] and Ronny Pini[*,†]

†*Department of Chemical Engineering, Imperial College London, South Kensington SW7 2AZ, United Kingdom*

‡*BP International Ltd, Chertsey Road, Sunbury-on-Thames TW16 7LN, UK*

E-mail: r.pini@imperial.ac.uk

Introduction

Associated supporting information for main text. Includes a more detailed illustration of the experimental set-up, pure component CT numbers measured, information regarding error handling with X-ray CT result interpretation, additional solution characterisation results, the underlying assumptions for the fractional flow approach and the fractional flow application itself.

Detailed Experimental Setup

Figure S1 presents a detailed P&ID diagram for the experimental coreflooding set-up. Included are all valves and associated line sizing. Primary flow lines are PTFE whilst confining pressure lines are PEEK tubing rated to 27 bar and 275 bar respectively. Flow path valves are a combination of Hylok ball valves and Hylok check valves; valves connected to P-001 are Sitec. PSV-015 is a Swagelok spring loaded pressure relief valve.

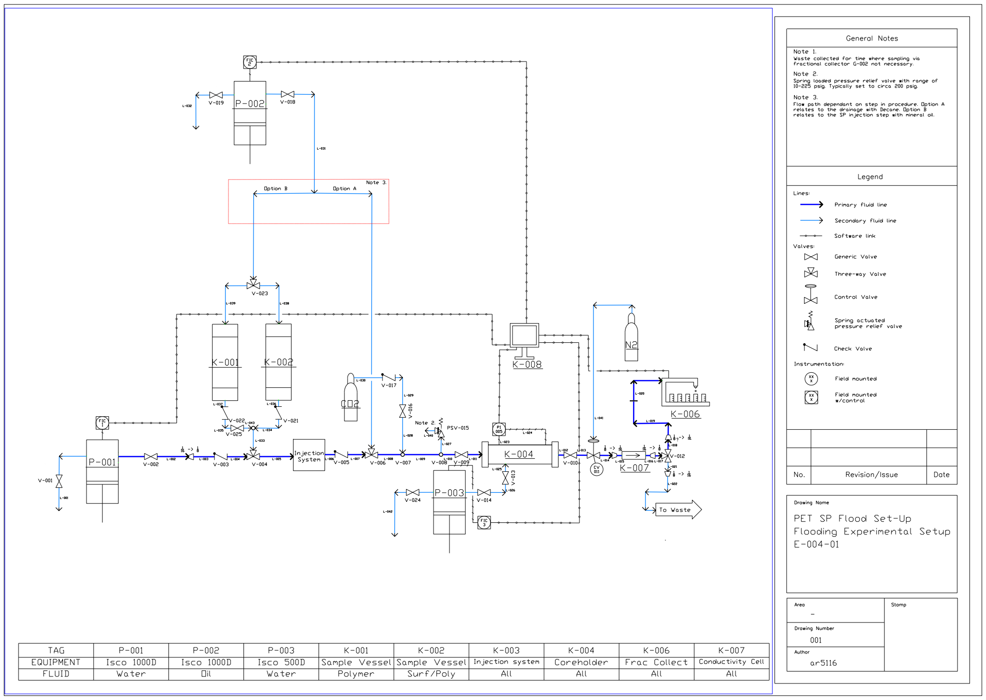

Figure S1: Full P&ID for experimental coreflooding set-up. Not shown here is the X-ray CT scanner in which the coreholder resides during experiments utilising direct imaging. Details of individual components are given in the main text.

Pure Component CT Numbers

Pure component CT numbers were calculated by taking scans of vials containing the relevant fluids placed within the coreholder. This was repeated for all experiments and as such, mean values and the associated 95 % confidence interval can be reported - shown in Table S1.

Table S1: Mean values and associated 95 % confidence intervals of pure component CT numbers from all experiments. For the aqueous phase, the CT number are split between surfactant A (SDBS/isobutanol mixture) and surfactant B (ALFOTERRA mixture) given the differing salinities.

CT_a	-834.4 ± 5.8 HU
CT_o	-140.2 ± 5.6 HU
CT_w^A	145.1 ± 4.2 HU
CT_w^B	145.4 ± 3.0 HU

Error Propagation and Correlation

To quantify and manage error an approach similar to that of Pini et al.[1] was used. For this, differences in repeated scans were calculated and this error was then propagated using equations derived from the propagation of variances.[2]

$$\sigma_f^2 = \sum_{n=1}^{p} \left(\frac{\partial f}{\partial x_n}\right)^2 \sigma_{x_n}^2 \tag{1}$$

For porosity this is done as follows,

$$\phi = \frac{\mathrm{CT_{wc}} - \mathrm{CT_{ac}}}{\mathrm{CT_w} - \mathrm{CT_a}} = \frac{a-b}{\mathrm{const}}$$

$$\begin{aligned}
\sigma_\phi^2 &= \sigma_a^2 \left(\frac{1}{\mathrm{const}}\right)^2 + \sigma_b^2 \left(\frac{-1}{\mathrm{const}}\right)^2 \\
&= \sigma_{\mathrm{CT}}^2 \left(\frac{2}{(\mathrm{const})^2}\right) \\
&= \sigma_{\mathrm{CT}}^2 \frac{2}{(\mathrm{CT_w} - \mathrm{CT_a})^2}
\end{aligned}$$

yielding

$$\sigma_\phi = \frac{\sigma_{\mathrm{CT}}\sqrt{2}}{(\mathrm{CT_w} - \mathrm{CT_a})} \tag{2}$$

Similarly for saturation:

$$S_o = \frac{\mathrm{CT_{exp}} - \mathrm{CT_{wc}}}{\phi(\mathrm{CT_o} - \mathrm{CT_w})} = \frac{a-b}{c(\mathrm{const})}$$

$$\begin{aligned}
\sigma_{S_o}^2 &= \frac{1}{\mathrm{const}^2}\left[\sigma_a^2\left(\frac{1}{c}\right)^2 + \sigma_b^2\left(\frac{-1}{c}\right)^2 + \sigma_c^2\left(\frac{b-a}{c^2}\right)^2\right] \\
&= \frac{1}{\mathrm{const}^2}\left[\frac{2\sigma_{\mathrm{CT}}^2}{c^2} + \sigma_\phi^2\left(\frac{b-a}{c^2}\right)^2\right] \\
&= \frac{1}{(\mathrm{CT_o} - \mathrm{CT_w})^2}\left[\frac{2\sigma_{\mathrm{CT}}^2}{\phi^2} + \sigma_\phi^2\left(\frac{\mathrm{CT_{wc}} - \mathrm{CT_{exp}}}{\phi^2}\right)^2\right]
\end{aligned}$$

thus

$$\sigma_{S_o} = \frac{1}{(\mathrm{CT_o} - \mathrm{CT_w})}\sqrt{\left[\frac{2\sigma^2_{\mathrm{CT}}}{\phi^2} + \sigma^2_{\phi}\left(\frac{\mathrm{CT_{wc}} - \mathrm{CT_{exp}}}{\phi^2}\right)^2\right]} \tag{3}$$

The error from sequential scans was found to be both centred around 0 and following a normal distributions - characteristic of a random error - and are shown in Figures S2 and S3 (*top*) for the two dimensional and three dimensional representations respectively subject to four differing coarsening schemes. Additionally, as was done by Joss and Pini [3], we similarly investigated the presence of auto-correlation within the errors. This was achieved by comparing errors in a voxel in three dimensions, or cells in two dimensions, with that of their neighbours - results given in the lag plots from Figures S2 and S3 (*bottom*).

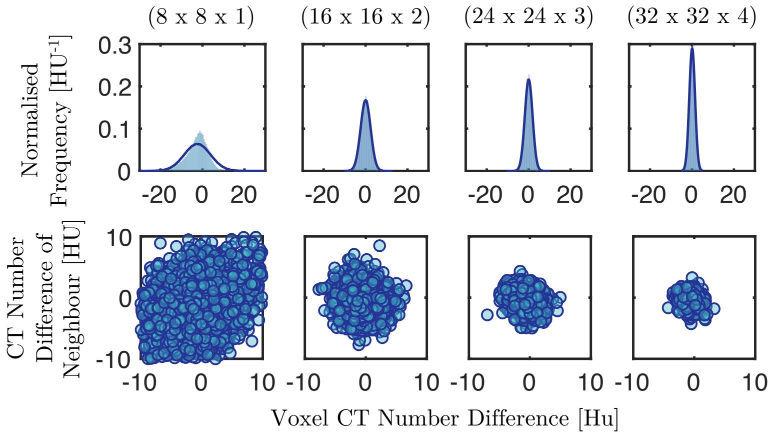

Figure S2: Error analysis for the two-dimensional representations in this work. Shown are the histogram of the voxel's random error from the X-ray CT scanning (*top*) and the associated lag plots of said error compared to neighbouring voxels (*bottom*). These are illustrated for four different super-sampling schemes – chosen for this work was the (16 x 16 x 2) voxel super-sampling, this yielded a voxel size of $(1.95 \times 1.95 \times 2)$ mm^3.

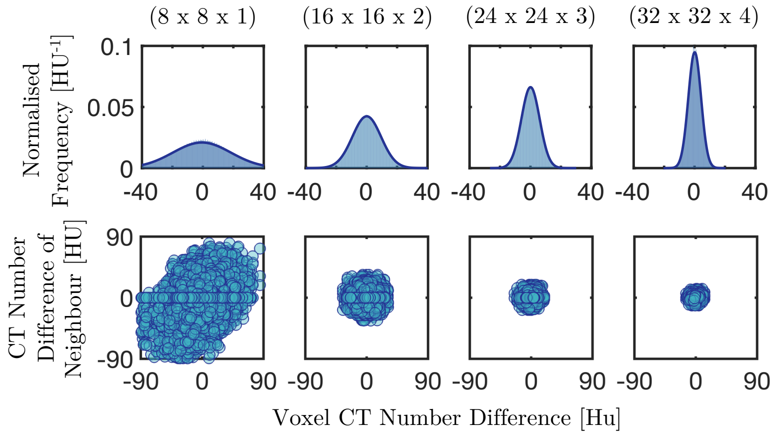

Figure S3: Error analysis for the three-dimensional representations in this work. Shown are the histogram of the voxel's random error from the X-ray CT scanning (*top*) and the associated lag plots of said error compared to neighbouring voxels (*bottom*). These are illustrated for four different super-sampling schemes – chosen for this work was the (24 x 24 x 3) voxel super-sampling, this yielded a voxel size of $(2.93 \times 2.93 \times 3)$ mm^3.

Lastly, applying Equations (2) and (3), the errors in porosity and saturation respectively are given in Table S2.

Table S2: Errors in porosity and saturation for differing coarsening schemes tested. Reported are both the errors associated with three-dimensional and two-dimensional reconstructions - first and second column under each voxel size considered respectively. For two dimensional images the chosen voxel size was $(1.95 \times 1.95 \times 2)\text{mm}^3$ and for the three dimensional images the chosen voxel size was $(2.93 \times 2.93 \times 3)\text{mm}^3$.

	$(0.98 \times 0.98 \times 1)\text{mm}^3$		$(1.95 \times 1.95 \times 2)\text{mm}^3$		$(2.93 \times 2.93 \times 3)\text{mm}^3$		$(3.9 \times 3.9 \times 4)\text{mm}^3$	
σ_ϕ	1.9×10^{-2}	6.3×10^{-3}	9.6×10^{-3}	2.4×10^{-3}	6.1×10^{-3}	1.9×10^{-3}	4.2×10^{-3}	1.4×10^{-3}
$\sigma_{\text{S}_\text{o}}$	0.28	0.091	0.138	0.035	0.088	0.027	0.0605	0.0201

Solution Characterisation Results

Figure S4 presents the phase test results for the surfactant formulations in a photographic form. As mentioned in the main text, based on the application of Huh's relationship, approximate IFT values of $1.5 \times 10^{-2}\,\mathrm{mN\,m^{-1}}$ and $\leq 1 \times 10^{-3}\,\mathrm{mN\,m^{-1}}$ for surfactant formulations A and B were calculated respectively

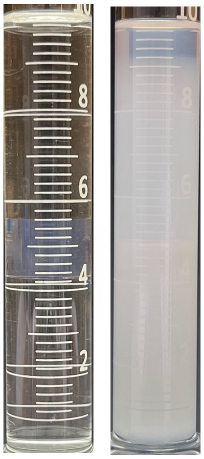

Figure S4: Photographic results of phase tests for both surfactant solutions at optimal salinity. Surfactant A refers to the SDBS/isobutanol solution whilst Surfactant B refers to the L-145-10s 90 solution. Corresponding optimal salinities were 3.7 %wt and 3.5 %wt NaCl.

Viscosity of injected surfactant/polymer solutions were mentioned to target 25 mPa s. The measurements for the two surfactant/polymer solutions are given in Figure S5.

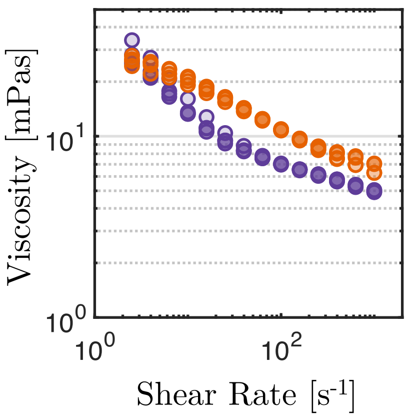

Figure S5: Viscosity measurements for surfactant/polymer solutions for both formulation A (SDBS/isobutanol), shown in purple, and formulation B (ALFOTERRA), shown in orange. Two samples and both forward and reverse shear sweep are illustrated. Despite the complex rheology, the results are consistent between samples.

Fractional Flow Theory Assumptions

- One dimensional flow
- Homogeneous porous media
- Incompressible flow
- Isothermal flow
- Effective porosity of conservative tracer is equal to rock porosity.
- Negligible dispersion
- No gravity or capillary forces present
- Uniform initial fluid distribution
- Non-competitive adsorption isotherm

Fractional Flow Theory Application - SP Floods

As outlined in the main text, an extended fractional flow approach was applied in order to highlight the difference in model capability of capturing internal dynamics based on the degree of self-similarity within the experiments. This was done by comparing two experimental extremes based on the sill value extracted from analysing the extent of overlap between consecutive self-similarity profiles.

The resulting constructions for the approach are shown in the Walsh diagrams in Figures S6 and S7 for the short and large core respectively.

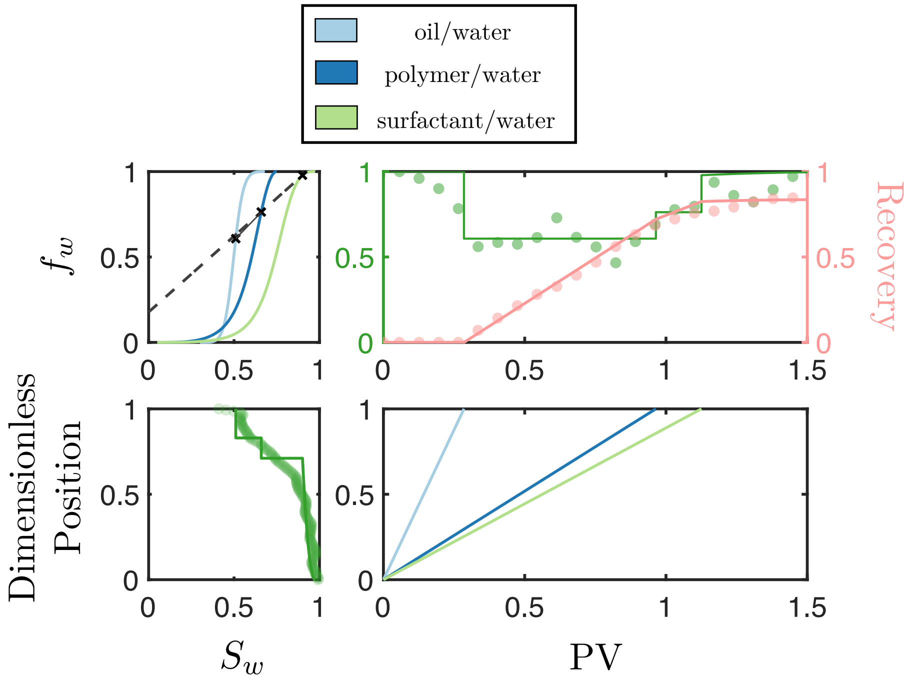

Figure S6: Walsh diagram for extended fractional flow approach applied to experiment 1 (small core). Shown here is the internal saturation profile corresponding to $\tau = 0.8$.

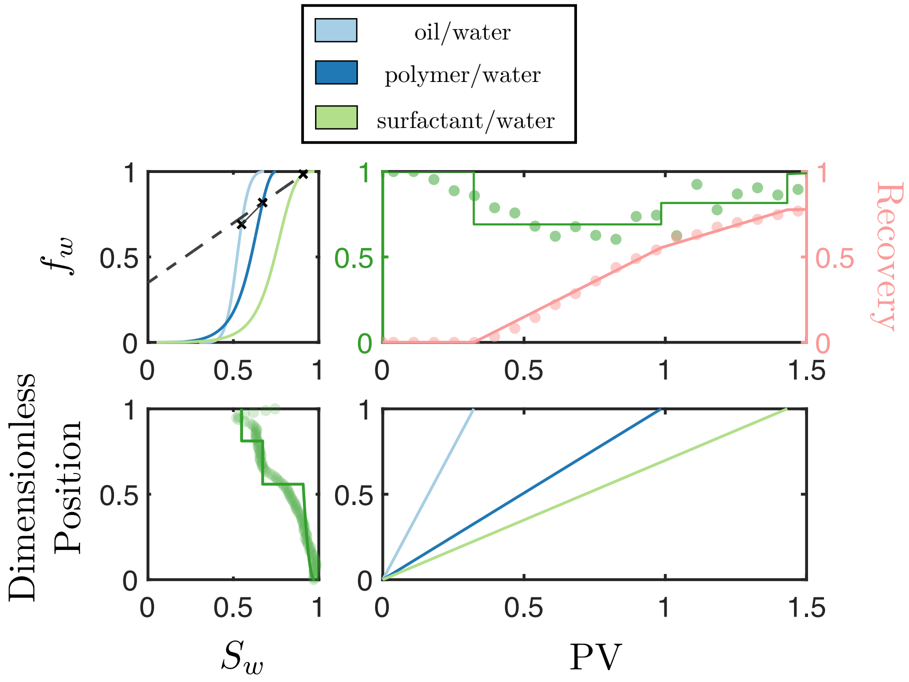

Figure S7: Walsh diagram for extended fractional flow approach applied to experiment 3 (large core). Shown here is the internal saturation profile corresponding to $\tau = 0.8$.

In both cases, the fluid and rock parameters necessary to build the fractional flow curves - top left illustrations - were altered as to provide a reasonable match to the outlet oil cut, and associated recovery, for the experiments - top right illustrations. Utilising the resulting fractional flow curves curves, predictions on the internal saturation profiles - bottom left illustrations - can be made utilising the associated front velocities - bottom right illustrations. The internal saturation profile prediction was repeated for numerous time-steps and quality of fits calculated and compared to the associated for the outlet to gauge its development and thus the applicability of the approach to predicting internal flow dynamics.

References

(1) Pini, R.; Krevor, S. C.; Benson, S. M. Capillary pressure and heterogeneity for the CO_2/water system in sandstone rocks at reservoir conditions. *Advances in Water Resources* **2012**, *38*, 48–59.

(2) Rouaud, M. *Probability, Statistics and Estimation: Propagation of Uncertainties in Experimental Measurement*; 2013.

(3) Joss, L.; Pini, R. Digital Adsorption: 3D Imaging of Gas Adsorption Isotherms by X-ray Computed Tomography. *121*, 26903–26915.
